# Activation of the Two-Component System LisRK Promotes Cell Adhesion and High Ampicillin Tolerance in *Listeria monocytogenes*

**DOI:** 10.3389/fmicb.2021.618174

**Published:** 2021-01-27

**Authors:** Hüsnü Aslan, Maiken Engelbrecht Petersen, Alberto De Berardinis, Maja Zacho Brunhede, Nasar Khan, Alberto Vergara, Birgitte Kallipolitis, Rikke Louise Meyer

**Affiliations:** ^1^Faculty of Natural Sciences, Interdisciplinary Nanoscience Center, Aarhus University, Aarhus, Denmark; ^2^Faculty of Veterinary Medicine, University of Teramo, Teramo, Italy; ^3^Department of Biochemistry and Molecular Biology, University of Southern Denmark, Odense, Denmark; ^4^Department of Biology, Faculty of Natural Sciences, Aarhus University, Aarhus, Denmark

**Keywords:** two-component systems, tcs, listeria monocytogenes, LisRK, adhesion, nanomechanics, antibiotic tolerance, persister

## Abstract

*Listeria monocytogenes* is a foodborne pathogen which can survive in harsh environmental conditions. It responds to external stimuli through an array of two-component systems (TCS) that sense external cues. Several TCS, including LisRK, have been linked to *Listeria*’s ability to grow at slightly elevated antibiotic levels. The aim of this study was to determine if the TCS LisRK is also involved in acquiring the high antibiotic tolerance that is characteristic of persister cells. LisRK activates a response that leads to remodeling of the cell envelope, and we therefore hypothesized that activation of LisRK could also increase in the cells’ adhesiveness and initiate the first step in biofilm formation. We used a Δ*lisR* mutant to study antibiotic tolerance in the presence and absence of LisRK, and a GFP reporter strain to visualize the activation of LisRK in *L. monocytogenes* LO28 at a single-cell level. LisRK was activated in most cells in stationary phase cultures. Antimicrobial susceptibility tests showed that LisRK was required for the generation of ampicillin tolerance under these conditions. The wildtype strain tolerated exposure to ampicillin at 1,000 × inhibitory levels for 24 h, and the fraction of surviving cells was 20,000-fold higher in the wildtype strain compared to the Δ*lisR* mutant. The same protection was not offered to other antibiotics (vancomycin, gentamicin, tetracycline), and the mechanism for antibiotic tolerance is thus highly specific. Furthermore, quantification of bacterial attachment rates and attachment force also revealed that the absence of a functional LisRK rendered the cells less adhesive. Hence, LisRK TCS promotes multiple protective mechanisms simultaneously.

## Graphical Abstract

Listeria monocytogenes activates the two-component system LisRK under stress, and becomes more adhesive and highly tolerant to ampicillin.

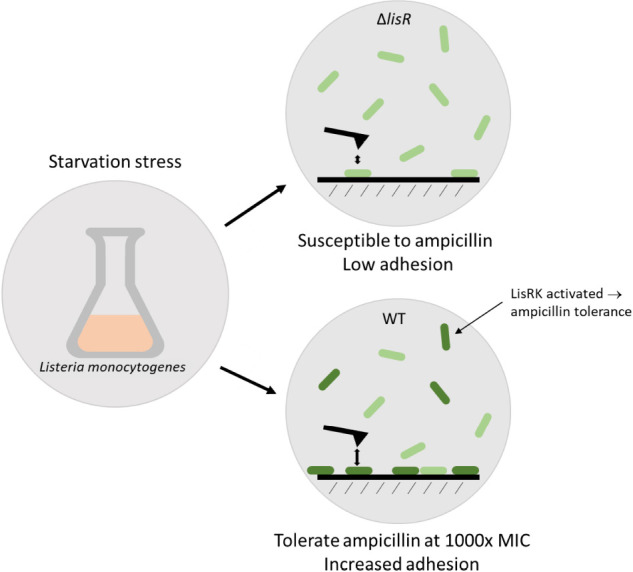

## Introduction

*Listeria monocytogenes* is a Gram-positive bacterium which causes listeriosis in susceptible individuals (Cossart, 2011). It is one of the most virulent foodborne pathogens with fatality rates exceeding even *Salmonella* spp. and *Clostridium botulinum* (Bintsis, 2017). Unlike most foodborne bacteria, it is capable of growth and reproduction in inhospitable environments under acidic or osmotic stress, and at cold temperatures as low as 0°C (Bucur et al., 2018).

One of *L. monocytogenes*’ survival strategies outside the host is to attach to surfaces and form a biofilm that protects it against environmental stressors (Stepanović et al., 2004; NicAogáin and O’Byrne, 2016). Biofilms are notoriously tolerant to antibiotics, and this tolerance is in part attributed to the presence of persister-cells in the biofilm. Persister-cells are non-dividing cells with a low metabolic activity that can revert to an active and dividing state under the right conditions. Bacteria can enter into a persister state due to starvation or stress that lead to arrested protein synthesis (Wood et al., 2013). The activation of stress responses and inactivation of cellular processes targeted by antibiotics allows the bacteria to tolerate and persist through exposure to very high concentrations of antibiotics, hence the term “persister-cells.” The molecular mechanisms that lead to persister formation are subject, and although some common mechanisms exist, there is some variation in how persisters form among different bacterial species. Likewise, the factors that trigger persister formation in response to stress will depend on how individual species detect and respond to such stresses.

**FIGURE 1:**
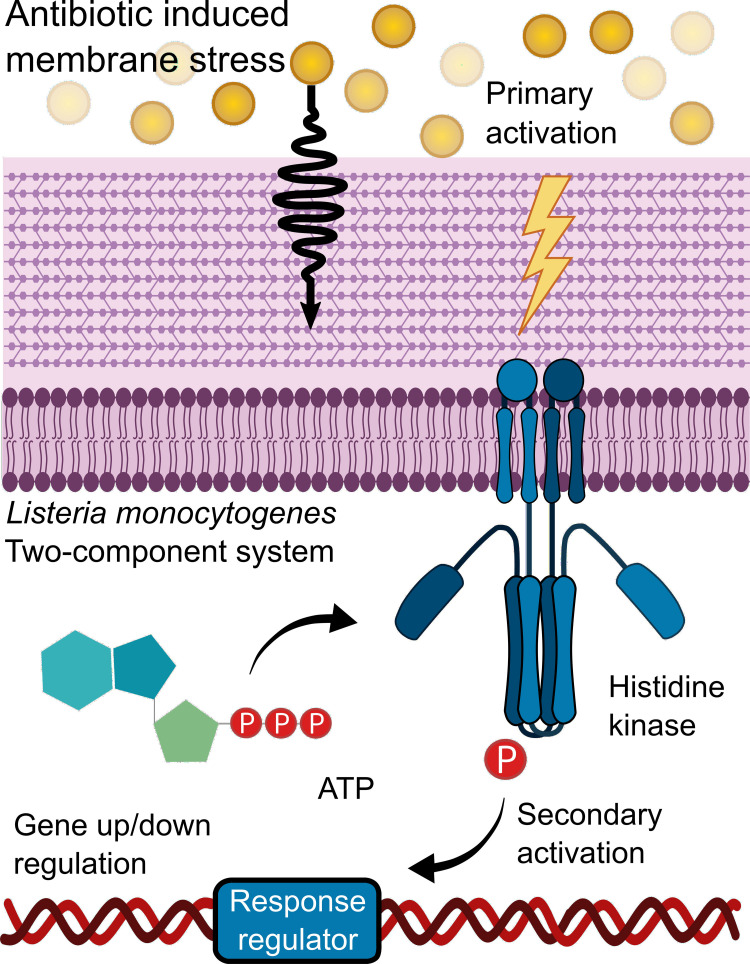
Two-component systems activation. An external environmental factor such as antibiotic-induced membrane stress leads to the primary activation of a sensor Histidine kinase which autophosphorylates. During the secondary activation, the phosphoryl group is transferred to a response regulator, thereby activating it. This leads to the response regulator acting as a transcription factor which regulates various genes.

*L. monocytogenes*’ adapt and survive in stressful environments due to its “environmental awareness” as it detects and responds to extracellular cues via two-component systems (TCSs), which regulate gene expression on cue as illustrated in Figure 1. Typically, a TCS is comprised of a transmembrane sensor histidine kinase and its cognate cytoplasmic response regulator. Upon signal induction from the environment, the histidine kinase autophosphorylates. Then the phosphoryl group transfers from His on the kinase to the N-terminal Asp residue on the response regulator. The activated regulator then acts as a transcription factor for the regulation of various genes (Casino et al., 2010). In the case of LisRK, which is the focus of this study, *lisK* encodes a transmembrane histidine kinase, and *lisR* encodes the cytoplasmic response regulator (Cotter et al., 1999). Depending on the environment, *L. monocytogenes* can vary its modes of survival, motility, and virulence even to the degree that it can switch between acting as a saprophyte or a pathogen (Freitag et al., 2009). Different activators can upregulate the virulence genes. For example, the PrfA thermoregulator UTR, an RNA thermometer, upregulates *L. monocytogenes*’ virulence genes at mammalian body temperature (Johansson et al., 2002), and the sigma factor Sigma B upregulates virulence genes in response to cues that are specific for the environment of the intestine (Camejo et al., 2009).

Little is known about how stressors trigger different modes of survival for *L. monocytogenes* outside a host. So far, 4 (LisRK, CesRK, LiaRS, and VirRS) out of the 14 TCS in *L. monocytogenes* (Glaser et al., 2001; Fritsch et al., 2011) are shown to partake in a decreased antibiotic susceptibility, allowing growth at slightly elevated concentrations of cell-wall targeting antibiotics in response to cell-envelope stress (Kang et al., 2015). We hypothesized that this response also results in elevated antibiotic tolerance, allowing the bacteria to survive temporary exposure to extreme antibiotic concentrations although they are unable to grow under these conditions. Some of the genes that are activated by LisRK induce changes of the cell envelope (Nielsen et al., 2012) and we therefore also hypothesized that such changes could affect cell attachment—the first step in biofilm formation (see Graphical Abstract). If LisRK activation promotes adhesion as well as antibiotic tolerance, this response to cell envelope stress ensures not only immediate protection but also long-term survival by initiating biofilm formation.

## Results

### LisRK Is Required for Persister-Like Ampicillin Tolerance

The effect of LisRK on antibiotic susceptibility was evaluated by quantifying the minimum inhibitory concentration (MIC) of commonly used antibiotics for listeriosis treatment against wildtype *L. monocytogenes* and a Δ*lisR* mutant (Table 1). The loss of a functional LisRK halved the MIC for ampicillin but not of the other antibiotics, which is in accordance with previous studies (Nielsen et al., 2012).

**TABLE 1:** Biocidal assays of various antibiotics against planktonic and different biofilm maturity levels of *L. monocytogenes* LO28 wild type (WT) vs. mutant lacking LisR two-component system (Δ*lisR*).

		WT	Δ*lisR*
MIC	Amp	0.5	0.25
	Vanc	1	1
	Tet	0.5	0.5
	Gen	4	4
MBC	Amp	16	0.5
	Vanc	16	16
	Tet	32	32
	Gen	8	8
MBEC (Treatment after 24 h biofilm growth)	Amp	>4,096	>4,096
	Vanc	>4,096	4,096
	Tet	1,024	1,024
	Gen	256	16
MBEC (Treatment after 1 h attachment)	Amp	>4,096	<4
	Vanc	1,024	1,024
	Tet	1,024	1,024
	Gen	32	16
MBEC (Treatment prior to attachment)	Amp	512	< 4
	Vanc	>4,096	1,024
	Tet	4,096	64
	Gen	<4	<4

**All results are in μg mL^–1^ from four biological replicates.**

Growth inhibition is not always sufficient to eradicate an infection, and it is therefore relevant to know the biocidal concentration of a drug. The minimal biocidal concentration (MBC) is the concentration of an antibiotic needed to cause at least a 1,000-fold decline in the number of viable cells over a 24 h incubation period. We show here that LisRK strongly affects *Listeria*’s ability to tolerate certain antibiotics, as MBC for ampicillin was 32-fold lower in the Δ*lisR* mutant compared to the wildtype. Again, the susceptibility to vancomycin, tetracycline and gentamicin was unaffected by the presence or absence of LisR (Table 1).

Activation of LisRK in the stationary phase triggered a response that increased ampicillin tolerance to a level that is comparable to what is observed in persister cells. Persister cells temporarily tolerate antibiotic exposure at very high concentrations, and the amount of persister cells in a culture is commonly quantified as the fraction of cells surviving antibiotic treatment at 10 × MIC for 24 h. The cells in this assay survived 24 h exposure to 32 × MIC ampicillin. We use the term “persister-like” ampicillin tolerance, as true persister cells must, in our opinion, display antibiotic tolerance to multiple antibiotics.

### Antibiotic Tolerance of Attached Cells Is Not a Response to Attachment

Bacteria display high antibiotic tolerance when growing as biofilm, mainly due to the presence of persister cells within the population (De la Fuente-Núñez et al., 2013). We therefore investigated LisRK’s effect on antibiotic tolerance of biofilms, which quantified as the lowest concentration of antibiotic needed to eliminate all viable cells in a biofilm, i.e., the minimal biofilm eradication concentration (MBEC). As expected, the antibiotic tolerance of biofilms was much higher in biofilms compared to planktonic bacteria (Table 1). LisRK did not affect the MBEC value of ampicillin, tetracycline and vancomycin, while the MBEC for gentamicin was lower in the mutant strain compared to the wildtype. As LisRK did not affect the inhibitory or biocidal concentration of gentamicin for planktonic bacteria, the difference observed in the MBEC value may be an indirect effect of differences in biofilm formation among the wildtype and mutant strain. However, this was not investigated further.

We were interested in understanding whether LisRK was involved in acquiring antibiotic tolerance at an earlier stage of biofilm formation, and we therefore measured the MBEC after only 1 h attachment. Remarkably, the antibiotic tolerance after 1 h of attachment was more than 300-fold higher for attached cells compared to planktonic cells for the antibiotics ampicillin, vancomycin, and tetracycline, while gentamicin only showed a 2–4-fold increased tolerance in the attached vs. the planktonic population. The increased tolerance to ampicillin was LisR dependent, but the tolerance to other antibiotics was not (Table 1). We did not consider the 2-fold difference in gentamicin tolerance for the wildtype and *lisR* mutant in this assay as being of significance.

**FIGURE 2:**
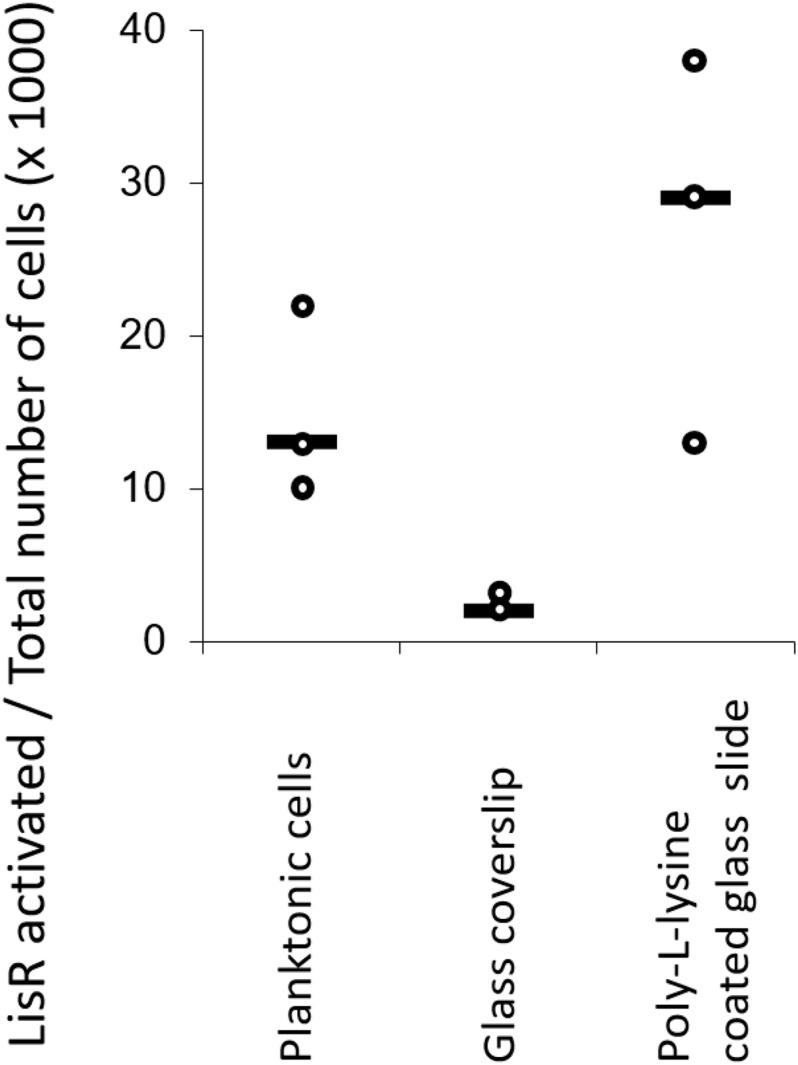
Attachment to surfaces with different chemistries does not activate LisRK. The fraction of cells with activated LisRK (i.e., with a positive GFP signal) was not significantly higher in attached vs. planktonic populations (Wilcoxon rank-sum test, *p* = 0.10 for planktonic cells vs. glass coverslip and *p* = 0.20 for planktonic cells vs. Poly-L-Lysine coated glass slide, *n* = 3 for all samples). LisR activation was quantified 3 h after attachment and subsequent flow. The line indicates the median.

It was intriguing that such a drastic increase in antibiotic tolerance to three antibiotics appeared to take place in less than 1 h after attachment. The change may occur as a response to attachment, possibly by activating LisRK and other TCS through mechanisms linked to surface sensing. After all, the high tolerance to ampicillin was only seen in the wildtype strain and not in the *lisR* mutant. To test this hypothesis, we created a reporter strain containing a plasmid with *gfp* fused to a LisR-inducible promotor. In an exponentially growing culture, LisRK was activated in 0.2% of the population, but this fraction increased to approximately 35% if LisRK was activated by exposure to sub-inhibitory antibiotic levels. If surface attachment activates LisRK, we expected a similar increase in the fraction of fluorescent cells. However, this was not the case (Figure 2). We investigated if LisRK different chemistries could activate LisRK in attached cells, but even attachment to surfaces coated with the antimicrobial Poly-L-lysine (PLL) did not trigger LisRK activation (Figure 2). In conclusion, the high ampicillin tolerance of attached cells was not caused by a LisRK-mediated response to the attachment.

Rather than originating from a response to attachment, we suspected that the apparent difference in antibiotic tolerance of planktonic and attached cells simply reflected methodological differences between the MBC and MBEC assays. The MBC assay quantifies the antibiotic concentration needed to kill 99.9% of bacteria in a planktonic culture during 24 h incubation. Quantification of viable cells is done by CFU enumeration after transferring a volume of the suspended bacteria to agar. However, a small amount of antibiotic is carried over to the agar plate along with the bacteria. Although it is diluted into the agar, it may still be sufficient to inhibit growth and result in underestimation of the number of viable cells, and thereby also the MBC value. Furthermore, the assay only reflects the antibiotic tolerance of the majority of the population and does not account for the properties of any sub-populations that comprise less than 0.1% of the population. Such sub-populations are not detected because their abundance is below the detection limit of the CFU enumeration method.

In contrast, the MBEC value reflects the antibiotic tolerance of the most tolerant cells in the population, no matter how few they are. The MBEC assay quantifies the antibiotic concentration needed to kill the entire population of bacteria in a biofilm. Biofilms are typically grown on the pegs of a peg-lid, which inserts biofilm-coated pegs into the wells of a 96-well plate with different concentrations of antibiotic in each well. After 24 h exposure, the peg lid is removed, washed, and transferred to a new plate with growth medium. A single surviving cell in the biofilm can then proliferate and repopulate the growth medium. In contrast to the MBC assay, the MBEC assay does not necessarily reflect the average properties of the entire population, but could potentially reflect the properties of a very small sub-population.

To determine if the discrepancy between the antibiotic tolerance of planktonic cells (MBC) and cells attached to a peg lid (MBEC) was caused by the different detection principles of the assays, we exposed planktonic bacteria to antibiotics for 24-h prior to attachment to the peg lid. The attached, pre-exposed cells were then transferred to fresh media to detect the presence of viable cells by measuring growth after 72 h incubation. This assay would probe the antibiotic tolerance of planktonic cells, using the detection principle of the MBEC assay. Despite some fluctuations in the results, these “planktonic MBEC” values were all highly elevated compared to MBC values, proving that the planktonic population contains a fraction of cells that are highly tolerant to ampicillin, vancomycin and tetracyclin, but not gentamicin. These highly tolerant cells were not identified in the MBC assay—either due to inhibited growth during CFU enumeration caused by the carry-over of antibiotics to agar plates or because the population of antibiotic-tolerant cells is below the detection limit (100 CFU mL^–1^) for the CFU assay. Presumably, the MBEC values reflect the antibiotic tolerance of persister-cells in the population, and it was confirmed once again that the high ampicillin tolerance depended on the presence of the two-component system LisRK.

### LisRK Activation Does Not Promote Persister-Like Tolerance to All β-Lactam Antibiotics

We proceeded to quantify how large the ampicillin-tolerant population was, and in doing so, we increased the ampicillin concentration to 1,000 × MIC. The LisRK-mediated tolerance to ampicillin ensured the survival of >7.8% of the cells when late-stationary phase cultures were exposed to ampicillin at 1,000 × MIC for 24 h, corresponding to an almost 20,000-fold increase in survival compared to the Δ*lisR* mutant (Table 2). We tested if the same applied to another β-lactam antibiotic cefuroxime, as previous studies had identified LisRKs involvement in cefuroxime tolerance. The fraction of cells surviving cefuroxime treatment was only 5-fold lower in the Δ*lisR* mutant, and the LisRK-mediated response did therefore not offer the same level of protection to this drug as it did to ampicillin.

**TABLE 2:** Fraction of cells (%) surviving 24 h antibiotic exposure at 500 μg mL^–1^.

	WT	Δ*lisR*
Cefuroxime	4.3% ± 2.0	0.8% ± 0.4
Ampicillin	7.8% ± 3.9	0.0004% ± 0.0003

### LisRK Activation Promotes Attachment

**FIGURE 3:**
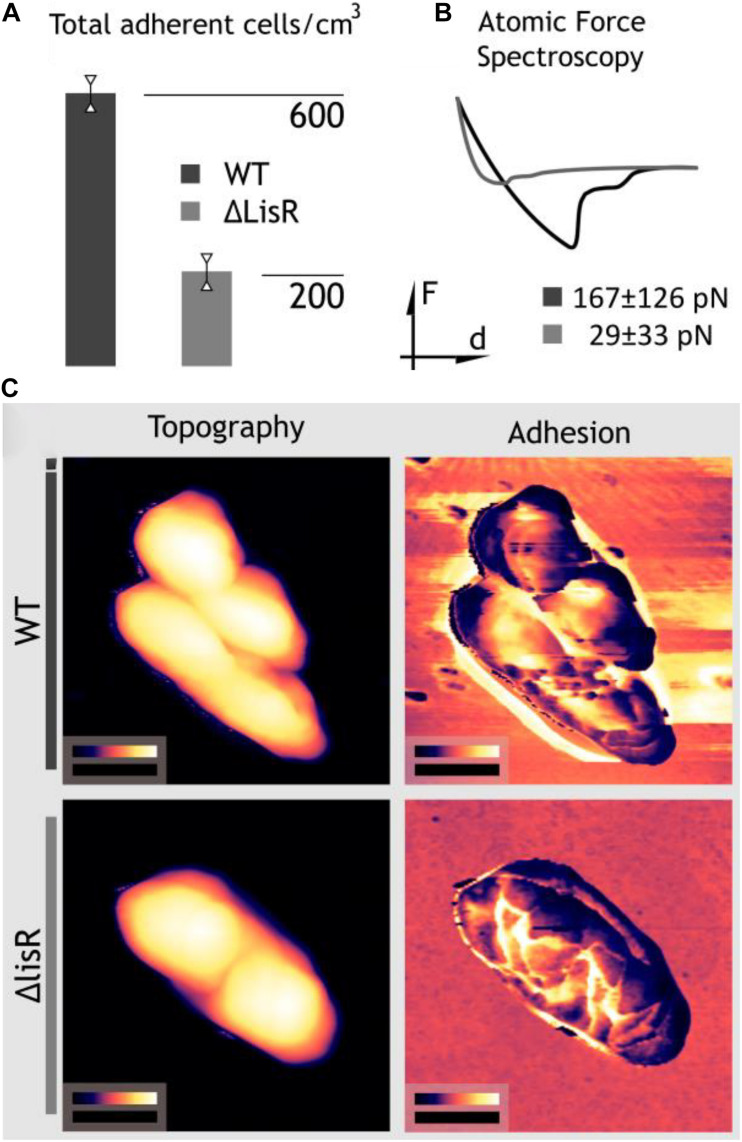
LisRK activation makes the cells more adhesive. **(A)** The average number of cells to a glass surface after 3 h continuous flow of a cell suspension (four replicates). A significant difference between the WT and *lisR* mutant was shown with Student’s *T*-test (*p* = 0.001). **(B)** Force spectroscopy for quantification of adhesion force of single *L. monocytogens* cells to a glass surface. The figure shows a typical force-distance curve, and average values ± standard deviations for 25 replicate cells is shown. **(C)** Nanomechanical mapping showing areas of topography images on the left and adhesion maps to the right. Black scale bars measure 500 nm; false color scales range from 0 to 370 nm for WT, 0–370 nm for ΔlisR cells in height images whereas in adhesion maps from 0 to 52.7 nN and 0–18.4 nN for Wt and ΔlisR cells, respectively.

Changes in the cell envelope affect how *L. monocytogenes* interacts with its environment, including solid/liquid interfaces that can be colonized to form biofilms. We therefore investigated if deletion of LisR led to cell envelope changes that affected bacterial attachment. By quantifying attachment under flow for 3 h, we show that the Δ*lisR* mutant was less capable of attachment, indicating that activation of LisRK makes the cells more adhesive (Figure 3A). There are many forces involved in bacterial attachment, and however, daunting it is to try to consider all the individual forces at play, it is possible to measure the net force directly by probing the cell envelope. We used atomic force spectroscopy to collect force-distance curves that quantify and average the net forces in the attachment of individual *L. monocytogenes* cells to a glass surface. The net attachment force was 5-fold higher for the wildtype compared to the Δ*lisR* mutant (Figure 3B), which explains the greater attachment rate under flow.

In order to understand the source of the increased forces, we must investigate deeper. The extended DLVO theory (named after Derjaguin, Landau, Vervey, and Overbeek) (Hermansson, 1999) provides one such explanation by describing the bacterial adhesion as:

$$\Delta {\text{G}}^{\mathrm{adh}}=\Delta {\text{G}}^{\mathrm{vdW}}+\Delta {\text{G}}^{\mathrm{dl}}+\Delta {\text{G}}^{\mathrm{AB}}$$

Where, ΔG^vdW^, ΔG^dl^, and ΔG^AB^ note the classical van der Walls interactions, double layer interactions, and acid-base interactions, respectively. Here the net adhesion force is described as the combination of long and short-range forces which are attractive like most van der Walls interactions or repulsive like the Coulomb interactions from the overlap between the electrical double layer of the cell and the substrate surface. Attractive hydrophobic forces and repulsive hydration effects are intended to be described by the acid-base component of the equation, which is a null term for when the cell is far away from the surface. The distance dependency of the components and the consideration of ΔG^AB^ only in short distances provide a good explanation for the strengthening of cell-substrate interactions as the cell approaches to contact. However, this model does not account for the specific binding interactions, re-construction of the cell envelope, synthesis and secretion of sugars, polymers, proteins, and eDNA for the creation of a slimy polysaccharide capsule and eventually, the extracellular matrix and multicellular complex is known as the biofilm.

In order to investigate this angle, we executed dynamic nanomechanical mapping with simultaneous topography imaging using AFM (Zhang et al., 2014). Topography images in Figure 3C show no apparent structural change while the adhesion maps visualize the increased adhesiveness of the cell surface and the formation of a sticky zone around the cells. The nanomechanical mapping thus indicates that activation of LisRK triggers the production of adhesive cell-surface components.

## Discussion

This study investigated the role of the TCS LisRK in important survival mechanisms for *L. monocytogenes*, namely antibiotic tolerance and adhesion to abiotic surfaces. Previous research has shown that TCS can promote biofilm formation, and examples of such involvement exist in a broad range of pathogens including several streptococci, *Acinetobacter baumanii*, *Escherichia coli*, *Salmonella enterica*, and *Pseudomonas aeruginosa* (Tierney and Rather, 2019). In the latter, TCS regulate the production of adhesive surface appendages such as pili, while other TCS are activated upon adhesion and promote polysaccharide production to secure the transition from reversible to the irreversible attachment (Mikkelsen et al., 2011). We observed that *L. monocytogenes* cells became less adhesive if LisR was lacking, and LisRK therefore affects the initial step in biofilm formation. However, the cell envelope changes responsible for this effect remain unknown and should be explored further to complete the picture of how *Listeria* modulates its cell envelope in response to stress.

The antibiotic tolerance of persister-cells is a key challenge in treating infections, as persister-cells resume activity and proliferate after antibiotic treatment is completed. However, much remains to be discovered to achieve a comprehensive view of what triggers persister-cell formation, and which sensing, signaling, and response mechanisms are involved in the transition to persister-cells. We show here that that the TCS LisRK is needed to achieve persister-like tolerance to the β-lactam antibiotic ampicillin, but not to other antibiotics tested.

In quantifying the antibiotic tolerance, we discovered an intrinsic flaw in the MBC assay for quantification of antibiotics’ biocidal effect. The transfer of antibiotics with planktonic suspensions to agar plates for CFU enumeration is sufficient to inhibit the growth on agar and thereby underestimate the CFU count and the MBC value. This is particularly problematic for drugs with a biocidal concentration that is much higher than the inhibitory concentration. Furthermore, the MBC value does not detect the antibiotic tolerance of any antibiotic-tolerant sub-populations, if their abundance is below the detection limit for CFU enumeration. We found that planktonic populations contained cells that tolerated more than 300-fold higher levels of ampicillin, vancomycin, and tetracycline, compared to the MBC values identified in the standard assay. This flaw in MBC assay can be overcome by washing cells before transfer to agar, and by improving the detection limit of the CFU enumeration. These methodological challenges must be considered when drawing conclusions from published MBC values.

Among the antibiotics tested, LisRK’s role appeared to be specific to ampicillin tolerance. The antibiotics were chosen based on their different modes of action. The β-lactam antibiotic ampicillin inhibits cell wall synthesis by binding to penicillin-binding proteins (transpeptidases) and preventing peptidoglycan crosslinking. The glycopeptide vancomycin also inhibits peptidoglycan crosslinking, but by binding D-Ala-D-Ala in the peptide. Tetracycline and the aminoglycoside gentamicin both inhibit protein synthesis by binding to the 30S ribosomal subunit and blocking t-RNA binding. We expanded the investigation to also include another β-lactam antibiotic, cefuroxime, due to previous reports of LisRK’s effect on the cefuroxime MIC for *L. monocytogenes*. While LisRK did affect cefuroxime tolerance, it was far less than its effect on ampicillin tolerance. After exposure to 1,000 × MIC, the survival rate of the wildtype strain compared to the Δ*lisR* mutant was 5-fold higher in the cefuroxime treatment, and 20,000-fold higher in the ampicillin treatment. Ampicillin is a 3rd generation penicillin while cefuroxime is a 2nd generation cephalosporin. Both antibiotics bind to the penicillin-binding proteins (PBPs) responsible for crosslinking peptidoglycan (Blumberg and Strominger, 1974). However, different β-lactam antibiotics do not necessarily bind to the same PBPs. Previous studies have linked LisRK and another TCS (CesRK) to the regulation of PBP expression that affected resistance to cephalosporins (Cogan et al., 2002; Cotter et al., 2002; Nielsen et al., 2012). These two TCS also affect each other, and CesR-regulated genes are upregulated more strongly in the Δ*lisR* mutant (Gottschalk et al., 2008; Nielsen et al., 2012). Perhaps the increased tolerance to specific β-lactam antibiotics in the Δ*lisR* mutant reflects a shift in the repertoire of transpeptidases being expressed, and the mechanism behind the change may not exclusively involve LisRK. Alternatively, increased antibiotic tolerance may come from changes in the cell envelope that affects the antibiotic’s access to its target. Several TCS trigger changes to the cell envelope in response to stress (Jordan et al., 2008; Fritsch et al., 2011; Nielsen et al., 2012) and these changes are not fully characterized.

TCS have been implicated in elevated antibiotic tolerance in other organisms through upregulation of antibiotic-degrading enzymes, regulation of transport proteins, or modulation of the cell surface (Tierney and Rather, 2019). However, knowledge about their implication in persister formation is very limited and best known from *E. coli* where the TCS NtrBC affects the expression of proteins required for carrying out the stringent response under nitrogen starvation (Brown, 2019). However, previous reports do show that one of the regulatory proteins associated with LisRK’s transcriptional response is PhoP, which is a master regulator for persistence and virulence in many human pathogens (Gonzalo-Asensio et al., 2008; Arenas et al., 2013). Given that bacteria enter the persister state as a response to starvation or stress, it seems likely that TCSs responding to cell envelope stress or metabolic imbalance could take part in persister formation. The highly selective antibiotic tolerance observed in our study shows that LisRK is not linked to persister formation *per se* but to a persister-like tolerance to ampicillin through a more specialized mechanism. Our observations raise the question of whether the multi-drug tolerance of persister-cells involves a suite of mechanisms that are specific to different classes of antibiotics, or if a few global mechanisms provide tolerance to a collective of antibiotics. Whether the tolerance is to a narrow or broad spectrum of antibiotics, involvement of a TCS in persister-like antibiotic tolerance points to the TCS as a potential therapeutic target for infections that become chronic due to the antibiotic tolerance of persister-cells.

## Experimental

### Bacterial Strains and Culturing Conditions

The construction of the lisR mutants was done based on the previously published work (Gottschalk et al., 2008). In short, *Listeria monocytogenes* strain LO28 wild type and Δ*lisR* mutant were inoculated from single colonies grown in BHI (brain heart infusion) broth for 16 h at 37°C. *L. monocytogenes* LO28—pLhrC5-pNF8 was grown in BHI supplemented with 5 μg mL^–1^ erythromycin for 16 h at 37°C. In cultures where LisRK was activated by exposure to sub-lethal antibiotics, 1 μg mL^–1^ cefuroxime was added to the media 16 h at 37°C.

### MIC and MBC assays

All antibiotics were purchased from Sigma-Aldrich with the designated product codes as follows: ampicillin (A9518), gentamicin (G1264), tetracycline (T7660), and vancomycin (V1130). Antibiotic stock solutions were prepared to dissolve the antibiotic powder in milliQ water or phosphate-buffered saline (PBS), as described by CLSI guidelines (Weinstein, 2019), and stored at −20°C until use. Antibiotics stock solution were serially diluted in BHI, and 180 μl of each was transferred to four wells in a microtiter plate (NunclonTM surface, Nunc, cat. no.: 161093). Fresh cultures were prepared by diluting overnight cultures with fresh BHI to reach a concentration (OD600) corresponding to ca. 5 × 10^6 CFU mL^–1^. Four wells were used as a negative control (blank), whereas 20 μl inoculation culture was added to each of the other wells (one well per replicate culture), reaching a final concentration of 5 × 10^5 CFU mL^–1^ per well. The microtiter plates were then incubated at 37°C for 16 ± 2 h, and MIC was measured using a plate reader (PowerWave XS2, BioTek) after the incubation period. Finally, 10 μl of the suspension contained in each well with no apparent growth was spotted onto a BHI agar plate and incubated for 24 h at 37°C to assess the MBC. The MIC was determined as the lowest antibiotic concentration resulting in no growth in the well. The MIC was determined as the lowest antibiotic concentration resulting in now surviving cells in the well (detection limit 5 × 10^2 CFU mL^–1^). Analyses were carried out in four biological replicates.

### MBEC Assays

The MBEC was determined as follows: Overnight cultures of *L. monocytogenes* were diluted in fresh BHI to OD600 = 0.1, and 160 μl was transferred to the wells in a microtiter plate. A peg lid (Thermo Scientific, Nunc-TSP, cat. no.: 445497) was inoculated by inserting it into this plate and incubating at 37°C for 1 h allowing planktonic cells to attach. For MBEC analysis on biofilms, the peg lid was then transferred to a new microtiter plate containing 160 μl/well of BHI, and incubated in a zip-lock bag at 37°C with 50 rpm of orbital shaking for 24 h to grow and form a biofilm. For MBEC analysis of attached cells, antibiotic exposure was commenced directly after the 1 h attachment step. The peg lid was then transferred to a plate containing a dilution series of antibiotics and incubated for 24 h. Detection of viable cells on the peg was then determined by washing the pegs twice by insertion in microtiter plates with 180 μl/well sterile PBS and transferring the peg lid to a “recovery plate” with 160 μl/well fresh media, sonicated (Ultrasonic Cleaner, VWR) for 10 min, and then incubated at 37°C for 72 h. MBEC was determined as the lowest antibiotic concentration leading to complete eradication of the biofilm as determined by the absence of growth in the recovery plate, measured by absorbance at 600 nm.

Another MBEC assay was conducted, in which the planktonic population was exposed to antibiotics for 24 h before inserting the peg lid for 1 h to allow attachment of the exposed population and proceeding with the washing and recovery steps in the MBEC assay as described above. This analysis addresses the antibiotic tolerance of the planktonic bacteria before attachment but uses the detection principle of the MBEC assay. Analyses were carried out in four biological replicates.

### Survival of Biocidal Antibiotic Exposure

Three replicate overnight cultures (inoculated from separate colonies) of *L. monocytogenes* WT, and Δ*lisR* were adjusted to OD_600_ = 1 in BHI containing 500 mg mL^–1^ ampicillin or cefuroxime, corresponding to approximately 100 × MIC for cefuroxime and 5,000 × MIC for ampicillin. Samples were collected for CFU enumeration immediately before addition of antibiotics, and after 24 h. Cells were centrifuged (10,000 × g, 7 min) and washed in sterile PBS twice before serially diluted and plated on BHI agar. CFU was counted after 48 h incubation at 37°C.

### Flow Cell Experiments

Overnight cultures of *L. monocytogenes* L028 WT and Δ*lisR* strains were adjusted to OD_600 nm_ = 0.1 by dilution in BHI and transferred to 60 ml syringes, mounted on a syringe pump and connected to a 6 channel flow cell (80606, IBIDI). The flow cell was inoculated for 1 h, at a flow rate of 20 ml h^–1^. The flow cell was subsequently washed with PBS for 10 min, at a flow rate of 20 ml h^–1^ before imaging of attached cells by phase-contrast optical microscopy (40× objective, Axiovert A1, Zeiss). Quantification of attached cells cm^–2^ was performed by image analysis in Fiji, ImageJ software. Analyses were carried out in 3 biological replicates with 10 images acquired from each replicate. The average density of attached cells was compared by Student’s *t*-test.

### Investigation of LisRK Activation on Various Surfaces

To examine LisRK activation, a reporter system constructed by fusing the promoter for the LisR-regulated gene *lhrC5* (Sievers et al., 2014) with gfp-mut1 in the plasmid, which contains erythromycin resistance. The *lhrC5* promoter sequence was amplified from *L. monocytogenes* LO28wt using Phusion DNA Polymerase (M0530, New England Biolabs) in a BioRad T 100 Thermal Cycler with the settings: 30× (15 s at 98°C, 15 s at 57°C and 15 s at 72°C). Forward primer: 5′-GGGGGAATTCGAAAAATAGGATTGCAGAAAAGC-3′ re- verse primer: 5′-GGGGGGATCCGCTTATACATATAATAACTT TTCTAAAA-3′. After amplification, the fragment was purified with PCR DNA and Gel Band Purification Kit (28903470, GE Heathcare) and digested with FastDigest *Eco*RI and FastDigest *Bam*HI (FD0274 and FD0054, Thermo Scientific) for 30 min at 37°C with and additional subsequent purification. The fragment was ligated into the pNF8 plasmid (Fortinea et al., 2000) overnight at 17°C using T4 DNA Ligase (EL0011, Thermo Scientific). The pNF8-*lhrC5* construct was transformed into chemically competent *L. monocytogenes* LO28 wt through electroporation at 2.5 kV, 25 μF, 200 ohm, 4.5 ms with subsequent incubation at 30°C for 2 h followed by growth on BHI agar plates with 5 μg mL^–1^ erythromycin for selection. This GFP reporter strain of *L. monocytogenes* was grown at 37°C, and 180 rpm shaking in BHI broth having 5 μg mL^–1^ erythromycin. Early exponential growth phase (OD_600_ = 0.10–0.30) was selected to examine LisR activation since LisR gets readily activated in the stationary phase. The flow cell was filled with 37°C BHI, and the cultures were then introduced to the flow cell setup by injecting 1 mL of the exponential phase culture into the flow cell. The culture was left to attach to the flow cell for 15 min before the flow was started at a flow rate of 1 mL h^–1^ for 3 h. The attached cells were stained with 25 μM SYTO 60 Red Fluorescent Nucleic Acid Stain (S11342 Invitrogen) and imaged in a Zeiss Confocal Laser Scanning Microscope. Images of minimum 600 cells were obtained per replicate, and total cell number and the total number of LisR active cells were collected by counting each cluster of pixels larger than three pixels in Fiji ImageJ. The attachment was quantified on standard glass coverslip (C9056 Sigma-Aldrich), positively charged glass slide (SuperFrost Ultra Plus, Thermo Fisher Scientific), and Poly-L-lysine (P4832 Sigma-Aldrich) coated glass slide. Each surface was washed before coating and introducing to bacteria by sonicating 10 min in 96% ethanol and 10 min in sterile MilliQ Ultrapure Water. Analyses were performed on four biological replicates.

### Atomic Force Microscopy

The 3D real space topographic measurements of bacteria with nanoscale spatial resolution coupled with simultaneous nanomechanical mapping were conducted using atomic force microscopy (DimensionIcon, Nanowizard IV, Bruker, United States). We have chosen probes (ScanAsyst, Bruker) which were sensitive enough (*k* = 0.4 N m^–1^) not to damage the soft cells and sharp enough (*r* = 21 nm) to acquire high-resolution maps. Experiments were conducted in room conditions. The cells in PBS were drop-casted on positively charged glass slides and left to adhere for 5 min then the surface was washed to remove unattached bacteria, and the PBS media was sucked from the surface with a tissue paper. The cells were then immediately imaged at 512 × 512 pixel resolution and 1 Hz scan rate.

In addition to imaging/nanomechanical mapping, force spectroscopy was done over a large number of samples to get a quantitative view of adhesion forces. The force-distance curves were then averaged, and the nominal fits to the averages are displayed. The collected AFM data was corrected to remove tilt, and obvious artifacts if any, using Gwyddion [Nečas, David, and Petr Klapetek. “Gwyddion: an open-source software for SPM data analysis.” Open Physics 10.1 (2012): 181-188] open source scanning probe image processing software. A total of 25 cells were analyzed from three biological replicates of each strain. Up to 10 force-distance curves were measured on each cell.

## Data Availability Statement

The raw data supporting the conclusions of this article will be made available by the authors, without undue reservation.

## Author Contributions

HA, MB, AD, RM, and BK planned the project. HA, MB, MP, AD, and NK conducted experimental work under the supervision of RM, BK, and AV. HA and RM wrote the manuscript draft. All authors edited the manuscript.

## Conflict of Interest

The authors declare that the research was conducted in the absence of any commercial or financial relationships that could be construed as a potential conflict of interest.
